# Reduction in Cerebral Perfusion after Heroin Administration: A Resting State Arterial Spin Labeling Study

**DOI:** 10.1371/journal.pone.0071461

**Published:** 2013-09-10

**Authors:** Niklaus Denier, Hana Gerber, Marc Vogel, Markus Klarhöfer, Anita Riecher-Rossler, Gerhard A. Wiesbeck, Undine E. Lang, Stefan Borgwardt, Marc Walter

**Affiliations:** 1 University Hospital of Psychiatry, Basel, Switzerland; 2 Department of Psychology, University of Basel, Basel, Switzerland; 3 Department of Radiological Physics, Basel University Hospital, Basel, Switzerland; 4 Medical Image Analysis Centre, Basel University Hospital, Basel, Switzerland; Medical University of Vienna, Austria

## Abstract

Heroin dependence is a chronic relapsing brain disorder, characterized by the compulsion to seek and use heroin. Heroin itself has a strong potential to produce subjective experiences characterized by intense euphoria, relaxation and release from craving. The neurofunctional foundations of these perceived effects are not well known. In this study, we have used pharmacological magnetic resonance imaging (phMRI) in 15 heroin-dependent patients from a stable heroin-assisted treatment program to observe the steady state effects of heroin (60 min after administration). Patients were scanned in a cross-over and placebo controlled design. They received an injection of their regular dose of heroin or saline (placebo) before or after the scan. As phMRI method, we used a pulsed arterial spin labeling (ASL) sequence based on a flow-sensitive alternating inversion recovery (FAIR) spin labeling scheme combined with a single-shot 3D GRASE (gradient-spin echo) readout on a 3 Tesla scanner. Analysis was performed with Statistical Parametric Mapping (SPM 8), using a general linear model for whole brain comparison between the heroin and placebo conditions. We found that compared to placebo, heroin was associated with reduced perfusion in the left anterior cingulate cortex (ACC), the left medial prefrontal cortex (mPFC) and in the insula (both hemispheres). Analysis of extracted perfusion values indicate strong effect sizes and no gender related differences. Reduced perfusion in these brain areas may indicate self- and emotional regulation effects of heroin in maintenance treatment.

## Introduction

In heroin-dependent patients, acute and chronic exposure to heroin is associated with impairments in a range of cognitive processes, including impulse control dysfunction [1]. On the other hand, heroin, chemically known as diacetylmorphine (DAM), reduces the craving and stress response, accompanied by relaxation and euphoria [2], [3]. It is used as a maintenance drug in heroin-assisted treatment programs in clinical settings in several countries [4]. When heroin is injected, it very rapidly crosses the blood brain barrier, due to the lipophilicity of the acetyl groups [5]. DAM itself has a short half-life of less than ten minutes and is decarboxylated into 6-monoacetylmorphine (6-MAM), 3-monoacetylmorphine (3-MAM) and then into morphine [6]. While the metabolite 3-MAM has no effect on opioid receptors, both 6-MAM and morphine bind to the μ-opioid receptor and act as agonists. Pharmacokinetic modeling leads to the conclusion that 6-MAM is the main active metabolite during the acute effects after heroin administration [7]. The metabolite 6-MAM is unique to heroin and could underlie the intense rewarding effects and the addictive properties [5], [8]. Opioid receptors belong to the class of G-protein coupled receptors (GCPR); they enhance the probability of presynaptic GABA release, which is linked to both the physiological and the psychotropic effects [9], leading to analgesia, respiratory depression and reduced blood pressure [10], [11]. Psychotropic effects include euphoria, relaxation, excitement, pleasure, and drowsiness [12], with a rush and an euphoric phase [13]. The rush is described as lasting only seconds and providing a rapid release of inner tensions and craving. In contrast, the euphoric phase may last several hours in a steady state and is characterized by inner happiness, drowsiness and a feeling of distance from surrounding events [14].

However, the neurofunctional foundations of the effects mediated by heroin administration are not well understood. Pharmacological magnetic resonance imaging (phMRI) permits an in vivo examination of how psychotropic drugs affect neural network function in the brain [15]–[25]. In the present study, we have examined the acute effects of heroin, using an arterial spin labeling (ASL) magnetic resonance imaging (MRI) technique. ASL provides non-invasive and absolute quantification of cerebral blood flow (CBF), using magnetically labeled arterial blood as an endogenous contrast agent. This is an advanced and established imaging method to track acute drug effects. In contrast to blood-oxygen-level-dependent (BOLD), ASL perfusion has been shown to be highly reproducible over long time scales, due to the absolute quantification of CBF [26]. We have investigated CBF in a task free resting state during the euphoria phase (after one hour) and we expected that heroin administration could be related to altered perfusion in areas of the brain implicated in the regulation of emotions.

## Materials and Methods

### Design

This study is part of a randomized, placebo-controlled clinical trial that has been registered (http://clinicaltrials.gov; ID NCT01174927). Each patient was scanned twice, with a short interval between scans (mean 9 SD 3.8 days). On one day, the subjects received an injection of heroin or placebo (saline) before the scan and on the other after the scan. Patients received their regular morning dose of diacetylmorphine, corresponding to half of their daily dose. The order of heroin and placebo administration was randomized and patients were blinded to what they received. A study nurse administered the substance intravenously 20 minutes before the scanning session started. The ASL perfusion data were acquired one hour after injection (Mean = 69 min; SD = 11 min), corresponding to the euphoria phase during heroin condition.

Heroin-induced alterations in physiological parameters were assessed within the scanner by measuring heart rate, blood pressure and blood oxygen saturation every 5 minutes. The psychological effects of the applied substance were assessed by a 45-item Heroin Craving Questionnaire (HCQ) [27] and visual analogue scales (VAS) for intoxication, sedation, withdrawal, and strength of drug effects (range 0 to 10).

### Study sample

Fifteen (9 male) non-left-handed heroin-dependent patients (ICD-10 = F11.22) were included in the study. All subjects participated in a national program of standardized DAM (“heroin”)-assisted treatment (JANUS Basel, Switzerland). Participants were told to abstain from illicit drug use other than prescribed heroin for the duration of the study, from alcohol intake for 72 hours and from tobacco consumption for 2 hours before scanning. Illicit substances and medications were controlled by a urine test at each session. The exclusion criteria were a positive alcohol breathalyzer test, a history of significant medical problems or a major mental disorder (other than substance use disorders).

### Ethics Statement

All patients gave written informed consent and Basel Ethics Committee (EKBB, Switzerland, [http://www.ekbb.ch/]) approved the study. Patients were informed that they received their regular heroin dose (half of a daily dose) before or after the scan.

### Image acquisition

Imaging was performed on a 3 Tesla MRI scanner (Magnetom Verio, Siemens Healthcare, Germany) at Basel University Hospital. For high resolution anatomical data, a 3D T1-weighted scan (MPRAGE) was acquired with 1×1×1 mm^3^ isotropic resolution, repetition time (TR) of 2000 ms, inversion time (TI) of 1000 ms and echo time (TE) of 3.4 ms. For perfusion data, a pulsed ASL sequence [28] based on a flow-sensitive alternating inversion recovery (FAIR) spin labeling scheme [29] was applied, combined with modified Q2TIPS (TI periodic saturation) pulse preparation and a single-shot 3D GRASE (gradient-spin echo) readout [30]. For quantification of cerebral blood flow (CBF), a time series with 14 incremental TI (200 to 2800 ms in 200 ms steps) was acquired. For each TI, two images were acquired: one after slice-selective inversion (control image) and one after non-selective inversion (labeled image). The sequence parameters were: repetition time 3200 ms, echo time 12.7 ms and spatial resolution 4.6×4.6×4 mm^3^ (interpolated to 2.3×2.3×4 mm^3^).

### Image processing and analysis

Maps of quantified CBF (perfusion maps) were calculated from ASL raw data. Difference images of label and control images at different TI were derived and the time course was fitted to a model describing different stages of arterial blood passage, using in-house software, [28]. The resulting MHD files were converted to the NIFTI format with MedINRIA 1.9.2 (http://med.inria.fr).

**Figure 1:**
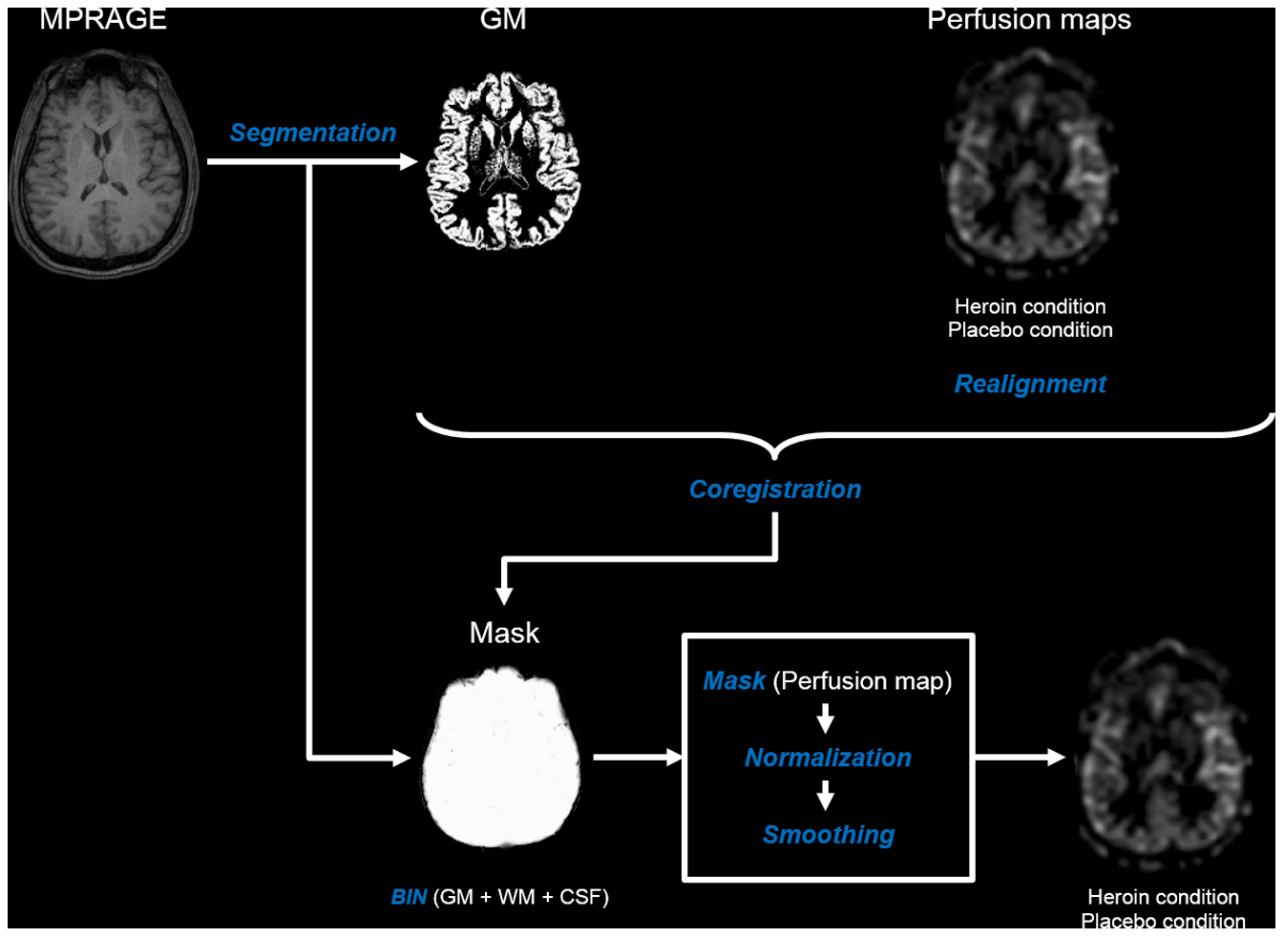
Overview of preprocessing steps at the subject level (SPM8). BIN: Binarization; CSF: Cerebrospinal fluid; GM: Gray matter; WM: White matter.

Further image processing and analyses were performed using Statistical Parametric Mapping (SPM8; Wellcome Department of Cognitive Neurology, London, United Kingdom). At the subject level, MPRAGE images were segmented to gray matter (GM) and intracranial tissue (ICT; sum of GM, white matter, and cerebrospinal fluid). Perfusion maps were reoriented to the standard direction. Both conditions were first realigned and then coregistered to segmented GM images, resulting in optimal MPRAGE coregistration. Then perfusion maps were masked with binarized ICT (binarization threshold: voxel intensity >0.1) to remove the extracerebral signal. The resulting perfusion maps were normalized to standard stereotactic MNI (Montreal Neurological Institute) space by applying the transformation parameters of normalized MPRAGE images. Finally normalized perfusion maps were smoothed using a 6 mm full-width-at half-maximum (FWHM) Gaussian kernel. All processed images were assessed with respect to quality and registration. See preprocessing steps in figure 1.

At the group level, a voxel-wise whole brain comparison between the heroin and placebo conditions was performed with a basic general linear model (GLM). A proportional scaling method in SPM8 was used to normalize each subject's perfusion maps. MNI coordinates of significant clusters were converted into Talairach space and labeled with the Talairach Client 2.4.3 (http://www.talairach.org/client.html).

### Statistical analysis

We performed a repeated measures (within-subject) analysis of variance (ANOVA) for whole brain analysis. Pre-processed heroin and placebo perfusion maps were used as input data. The following tests were applied: heroin < placebo (higher perfusion during heroin) and heroin > placebo (lower perfusion during heroin). Statistical significance was assessed at the cluster-forming threshold of p<0.01 (uncorrected). At the cluster level, only clusters with p<0.05 corrected for family-wise error (FWE) were considered significant [31]. We used the eigenvariate function in SPM8 to extract mean perfusion values within the significant clusters. Cohen's d was performed to calculate effect sizes of perfusion reduction. We used paired t-tests in SPSS Statistics (IBM SPSS Statistics; Armonk, NY: IBM Corp) to analyze differences in HCQ scores, VAS values and physiological parameters between the heroin and placebo conditions. To analyze demographic, clinical and perfusion differences (extracted values) between men and women, we used independent-samples t-tests. Pearson's r correlations were performed to assess the association of heroin dependence duration and extracted perfusion values.

## Results

### Demographic and clinical characteristics

The patients mean age was 41 years, they were heroin dependent for a mean of 21 years and had participated in the heroin-assisted treatment for a mean of 8 years. Mean daily heroin dose was 350 mg. Male and female patients didn't differ significantly in age (p = 0.10), duration of heroin dependence (p = 0.76), duration of heroin maintenance (p = 0.70) and daily dose of heroin (p = 0.44). Some patients also illicitly used other drugs, one third of patients had an additive substitution with a small dose of methadone. All participants were cigarette smokers. None of patients had abused alcohol. The patients' characteristics are described in table 1.

**Table 1:** Socio-demographic and diagnostic characteristics of the study sample.

Measurements	Subjects (n = 15)
Age (years), mean (SD)	40.9 (6.6)
Male gender, n (%)	9 (60.0)
Partnership, n (%)	6 (40.0)
Employment, n (%)	7 (46.7)
Age at first heroin use (years), mean (SD)	18.1 (3.0)
Duration of heroin dependence (years), mean (SD)	20.5 (7.7)
Duration of heroin maintenance (years), mean (SD)	7.0 (3.9)
Heroin dose (mg/day), mean (SD)	346.0 (173.4)
Methadone substitution, n (%)	5 (33.3)
Methadone dose (mg/day), mean (SD)	30.0 (10.0)
Cocaine use, n (%)	7 (46.7)
Cannabis use, n (%)	4 (26.7)
Tobacco use, n (%)	15 (100.0)

SD: Standard deviation.

### Physiological and psychological effects

**Figure 2:**
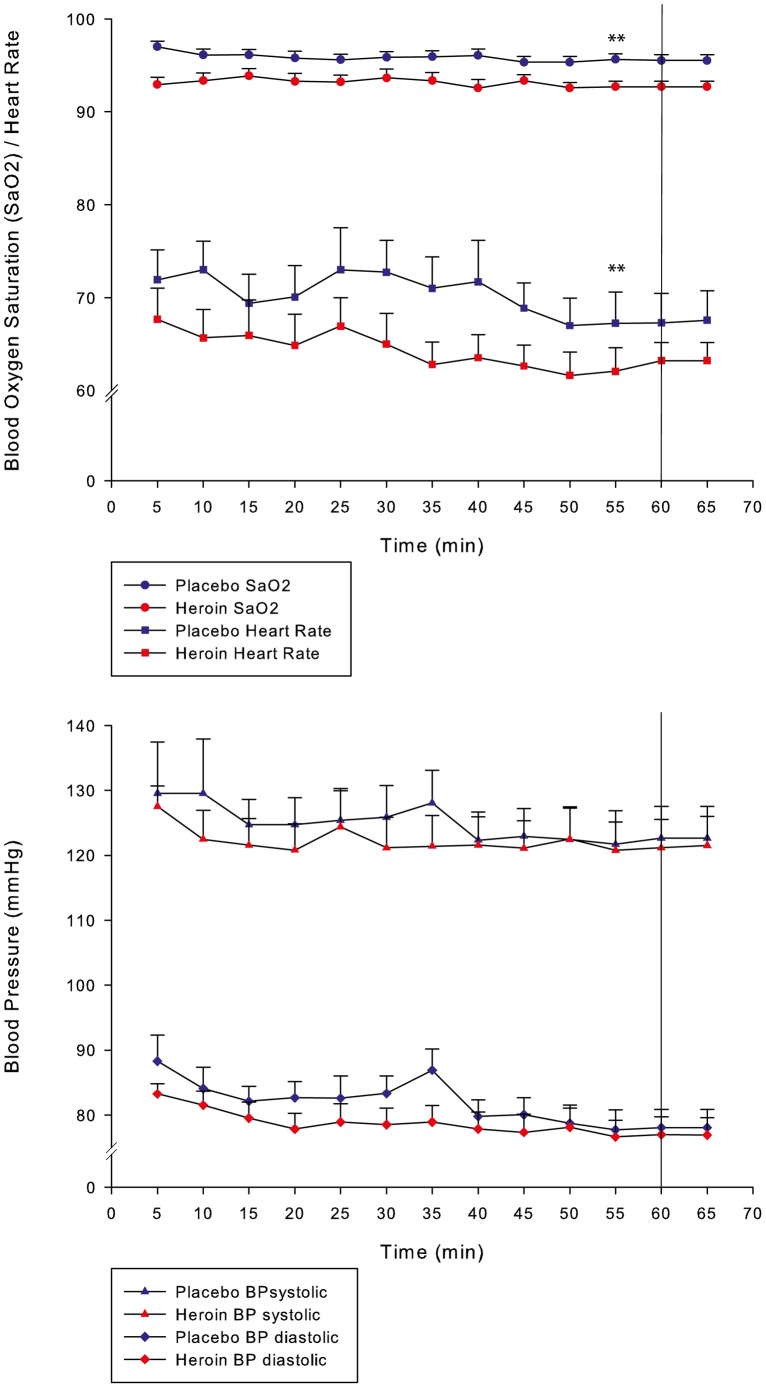
Physiological parameters after heroin and placebo administration. BP: Blood pressure; **: p<0.01.

In the heroin condition, patients had significantly lower heart rates and blood oxygen saturation, as shown in figure 2 (p<0.01). The differences remain significant at 60 minutes (p<0.05). There were no significant differences in the systolic and diastolic blood pressures, either in the mean values or at 60 minutes.

All patients correctly guessed the substance they had received before the scanning procedure. Perceived drug effects, feeling of intoxication and sedation, and relief from withdrawal were significantly higher during heroin condition than during the placebo condition (p<0.01). The desire to use heroin did not differ significantly between the two conditions. See the psychological effects of heroin in table 2.

**Table 2:** Psychological effects of heroin.

Measurements	Placebo	Heroin	p-value
HCQ: Desire to use heroin, mean (SD)	3.6 (0 to 14.5)	3.9 (0 to 15)	.849
HCQ: Intensions and plans to use heroin, mean (SD)	2.2 (0 to 9)	3.1 (0 to 9.5)	.005*
HCQ: Anticipation of positive outcome, mean (SD)	1.4 (0 to 6)	3.0 (0 to 14)	.056
HCQ: Relief from withdrawal/dysphoria, mean (SD)	1.0 (0 to 4)	3.1 (0 to 9)	.047*
HCQ: Lack of control over use, mean (SD)	3.3 (0 to 15)	2.0 (0 to 11.5)	.128
VAS: Intoxication, mean (SD)	1.1 (1.7)	3.7 (2.1)	.005*
VAS: Sedation, mean (SD)	1.7 (2.3)	3.3 (2.8)	.009*
VAS: Withdrawal, mean (SD)	3.7 (3.0)	0.4 (1.3)	.001*
VAS: Drug effect, mean (SD)	0.3 (1.0)	6.5 (2.9)	<.001*

HCQ: Heroin Craving Questionnaire; SaO_2_: Blood oxygen saturation; SD: Standard deviation; VAS: Visual Analogue Scale;

* : p<0.05.

### Brain perfusion characteristics

**Figure 3:**
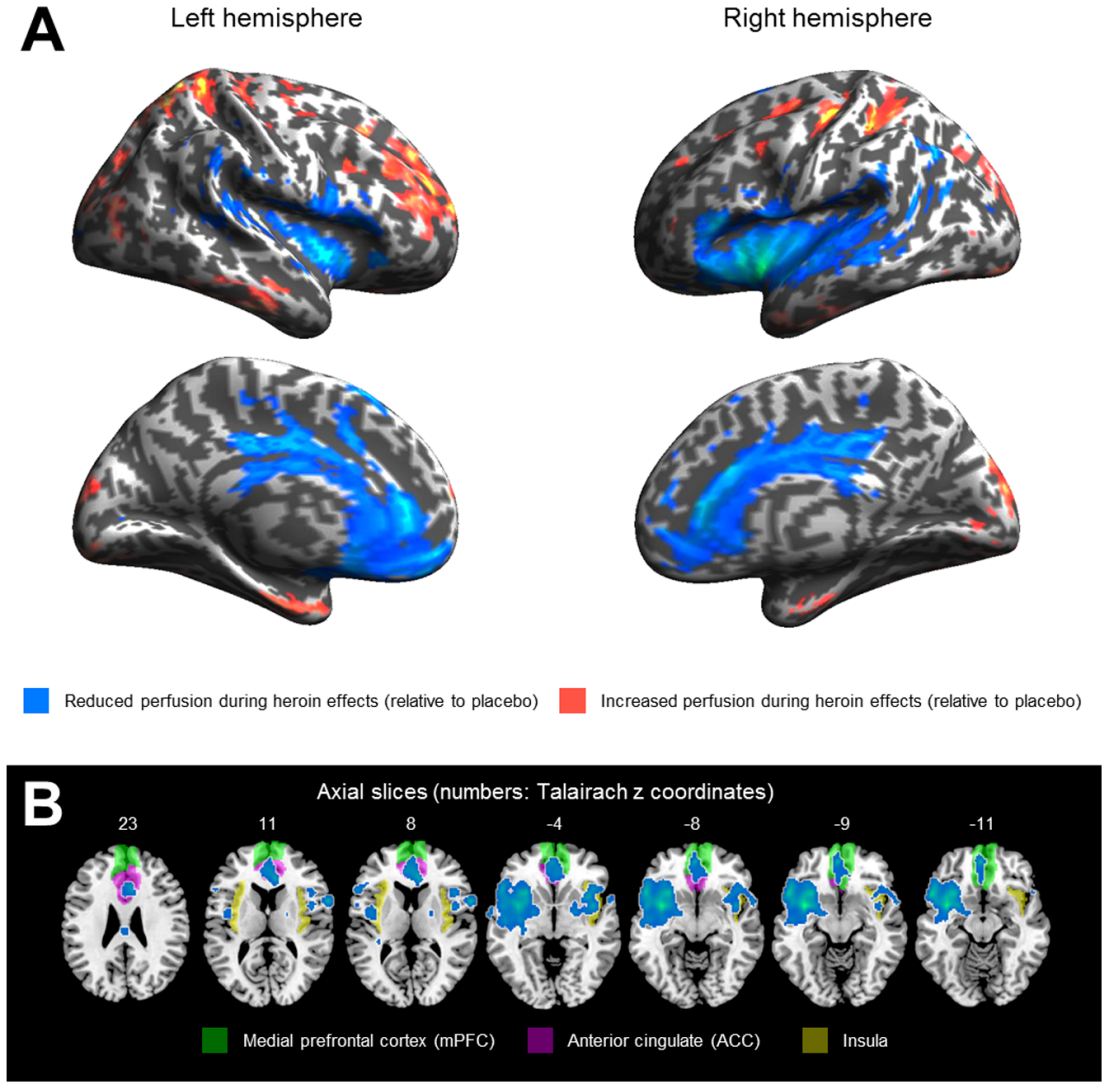
Altered regional perfusion in heroin in comparison to placebo condition. A) Relative hypo- (blue) and hyperperfusion (red) in heroin condition projected on an inflated brain (threshold: p<0.05). Hyperperfusion was not significant. B) Significant hypoperfusion (blue) in heroin condition (threshold: p<0.01; FEW corrected p<0.05). The z coordinate shows the position of each slice in reference to the Talairach atlas (see Table 3). The medial prefrontal cortex, the anterior cingulate cortex, and the insular cortex are shown in different colors, in order to clarify the spatial relationship to the significant clusters.

Compared to placebo, heroin administration was associated with relatively low perfusion in three main clusters (threshold: p<0.01). The first two clusters are within the area of the left and right insula (p<0.001 and p = 0.026; FWE corrected). The third cluster is in the area of the medial frontal cortex, with peaks in the anterior cingulate cortex (ACC) and the medial frontal gyrus (mPFC) (p<0.001; FEW corrected). No significant hyperperfusion (relative to placebo) was associated with heroin administration. Brain perfusion characteristics are shown in table 3 and figure 3.

**Table 3:** Effects of heroin on brain perfusion in contrast to placebo.

Area	Hemisphere	Talairach coordinates of	Cluster size	Cluster p-value	Cluster p-value
		cluster maximum (x y z)	(voxels)	(uncorrected)	(FWE corrected)
**Contrast: P(heroin) < P(placebo)**					
Insula (BA 13)	L	−38 3 −9	4590	<.001***	<0.001***
Superior Temporal Gyrus (BA 38)	L	−53 15 −11			
Insula (BA 13)	L	−30 17 −8			
Anterior Cingulate (BA 24)	R	8 33 8	2664	<.001***	<0.001***
Medial Frontal Gyrus (BA 11)	L	−2 50 −11			
Anterior Cingulate (BA 24)	R	8 23 23			
Precentral Gyrus (BA 44)	R	63 10 11	1379	0.001***	0.026*
Insula (BA 13)	R	36 13 −4			
Insula (BA 13)	R	40 4 −4			
**Contrast: P(heroin) > P(placebo)**					
None					

BA: Brodmann area; L: left; P: perfusion; R: right; WM: white matter;

* : p≤0.05;

*** : p≤0.001.

Each coordinate triple is a peak area of contrast intensity in one of the two significant clusters and refers to a specific area defined in the atlas of Talairach & Tournoux (1988).

Extracted mean perfusion values of significant clusters are shown in table 4. Comparison of heroin and placebo condition showed the following effect sizes in perfusion reduction: left insula d = 1.38, ACC/MPFC d = 0.93, right insula d = 0.82. Male and female patients didn't differ significantly in perfusion within all three clusters (p>0.05). Correlation analysis between extracted perfusion values and duration of heroin dependence showed no significant association (p>0.05).

**Table 4:** Regional perfusion values of significant clusters.

Region	Placebo	Heroin	Effect size
	(Mean/SD/CI 95%)	(Mean/SD/CI 95%)	(Cohen's d)
Cluster 1: left Insula	97.07/10.93/91–103	82.10/10.68/73–88	1.38
Cluster 2: right Insula	79.17/11.03/73–85	69.44/12.38/63–76	0.82
Cluster 3: ACC/MPFC	108.71/16.72/99–118	92.16/19.0/82–103	0.93

ACC: Anterior cingulate cortex; CI: Confidence interval; MPFC: Medial prefrontal cortex.

## Discussion

To our knowledge, this is the first study to measure selective heroin effects during the euphoria phase with an advanced phMRI method. We found that heroin administration in heroin-dependent patients is associated with a significant reduction in perfusion in the insula of both hemispheres, the anterior cingulate cortex (ACC) and medial prefrontal cortex (mPFC) with strong effect sizes. Whereas the insula and ACC play an essential role in emotional regulation and self-awareness [32], [33], the mPFC is known to be important in self-referential processing [34]. The extended limbic system and the mPFC are frequently involved in opiate abuse [35], [36].

A previous study examined the acute effects of heroin using functional neuroimaging [13]. Immediately after injection of heroin, there was increased perfusion in the left anterior cingulate gyrus, posterior cerebellar lobe, and right precuneus. After 15 minutes, heroin was associated with increased perfusion in the left superior frontal gyrus [13]. Another study was based on region of interest (ROI) analyses [37]. Eighty seconds after heroin injection, perfusion of the amygdalae was enhanced. In our study, we measured perfusion 60 minutes after heroin injection in a stable heroin maintenance treatment. In contrast to previous studies, we did not examine the acute effects in the first minutes after administration, but steady state effects before or after a heroin maintenance dose. Moreover, rather than 99mTc-HMPAO SPECT [13] or perfusion-weighted MR imaging (PWI) [37], our phMRI study employed ASL CBF quantification, which is highly reproducible and a well-established method to measure drug effects [26], [38], [39].

Interestingly, previous studies with the short acting opioid remifentanyl, which is used in anesthesia, showed that its acute effects leads to regional hyperpferusion in the ACC [15], [40] and in the insula [41]. These immediate effects of remifentanyl are in line with the perfusion findings during the rush phase of heroin [13]. Our findings, which represent more steady state effects of heroin-assisted treatment, support previous neuroimaging studies. After intramuscular morphine injection, glucose utilization was decreased by 10% in the whole brain, as well as in telencephalic areas [42]. In opioid-dependent subjects, prefrontal perfusion is generally reduced and the right-greater-than-left perfusion asymmetry, normally found in healthy probands, was found to be reversed [43]. Thus, we can argue that heroin-assisted treatment with regular and repeated heroin injection may reduce activity in specific brain areas.

The differences in perfusion, recognized by comparing the heroin and placebo conditions, may also be caused by hyperperfusion after placebo, which could be associated with withdrawal and craving. In general, placebo may induce craving and withdrawal symptoms in heroin-dependent patients [44]. However, all patients were under stable heroin maintenance treatment and did not show any acute withdrawal symptoms after the scanning procedure. We also found relatively low withdrawal levels and craving scores after placebo administration.

The present heroin-associated modulation of perfusion in prefrontal and extended limbic regions may also be related to the abnormal cerebral volumes reported in heroin-dependent patients [45], [46]. A possible direct connection between reduced perfusion during the acute effects of heroin and the reduced GM volume observed in heroin users could be that recurrent regional hypoperfusion may lead to metabolic impairment, affecting neural and glial function, and leading to a reduction in volume. This is in line with animal models of brain ischemia, which showed that a decline in CBF is associated with cognitive impairment and neural death [47], [48].

This reduced perfusion in these areas may underlay the feelings perceived by heroin users, such as relaxation and euphoria [49]. The mPFC and ACC are both parts of the cortical midline structures and the default mode network (DMN) [34]. The DMN has been postulated to be an intrinsic brain network, and is active when self-relevant mental stimulations are processed, such as non-goal-oriented thinking, autobiographical memory retrieval, envisioning the future, and conceiving the thoughts and perspectives of others [50], [51], [52]. The anatomy of the DMN can be divided into interacting subsystems, including the medial temporal lobe, which provides information from prior experiences in the form of memories and associations [53]. The mPFC acts as integrative component and provides flexible use of the temporal information during the construction of self-relevant mental stimulations.

The moderate number of participants limits our findings. Moreover, our patients were recruited from a population, which mainly consisted of individuals with long-standing polysubstance use. Although this problem is virtually inevitable when chronic heroin-dependent individuals are examined, it may have biased the results. Another limitation is the lack of a healthy control group and the use of an inactive placebo that could lead to withdrawal symptoms.

To conclude, our findings demonstrate that acute administration of heroin results in reduced brain activity within the left anterior cingulate cortex (ACC), the left medial prefrontal cortex (mPFC) and in the insula (both hemispheres). Reduced perfusion in these prefrontal and extended limbic areas may indicate self- and emotional regulation effects of heroin maintenance treatment.
